# He Pikinga Waiora: supporting Māori health organisations to respond to pre-diabetes

**DOI:** 10.1186/s12939-018-0904-z

**Published:** 2019-01-07

**Authors:** Angela Beaton, Carey Manuel, Jade Tapsell, Jeff Foote, John G. Oetzel, Maui Hudson

**Affiliations:** 1 Centre for Health and Social Practice, Waikato Institute of Technology, Hamilton, New Zealand; 2 Menzies Centre for Health Policy, University of Sydney, Sydney, Australia; 3 Poutiri Charitable Trust, Te Puke, New Zealand; 4 Department of Management, University of Otago, Dunedin, New Zealand; 5 School of Health Sciences, University of Canterbury, Christchurch, New Zealand; 6 School of Management, University of Waikato, Hamilton, New Zealand; 7 Faculty of Māori and Indigenous Studies, University of Waikato, Hamilton, New Zealand

**Keywords:** Māori health, Diabetes, Implementation science, Health equity, Indigenous

## Abstract

**Background:**

Type 2 Diabetes (T2D) is a common long-term condition affecting the health and wellbeing of New Zealanders; one in every four New Zealanders is pre-diabetic. Māori, the Indigenous people of New Zealand, are at an increased risk of developing pre-diabetes and T2D and there are significant inequities between Māori and non-Māori for T2D complications. The purpose of this study was to explore the questions of how the strengths of Māori heath organisations may be leveraged, and how the barriers and constraints experienced by Māori health organisations may be negotiated, for the benefit of Māori; and from a systems perspective, to identify strategic opportunities that may be considered and applied by Māori health organisations, funders and policy makers to respond more effectively to pre-diabetes and reduce health inequities between Māori and non-Māori.

**Methods:**

Utilising case study methodology, a range of data sources were triangulated including nine semi-structured interviews, documents, and a diabetes system map to identify possible strategic opportunities for key stakeholders to respond more effectively to pre-diabetes.

**Results:**

Key themes and possible actions to improve health outcomes for Māori with pre-diabetes include: (1) Recognising Māori health organisations as conduits for the community voice and influential partners in the community to effect change; (2) Strengthened partnerships with Māori health organisations for community benefit and to support measurable, evidence-based change and service delivery, particularly when Māori knowledge systems are viewed alongside a Western scientific approach; and (3) Intersectoral integration of health and social services to support provision of whānau-centred care and influence the social determinants of health and local environment.

**Conclusions:**

Māori health organisations are important actors in systems seeking to improve outcomes and eliminate health inequities. Support from funders and policy makers will be required to build on the strengths of these organisations and to overcome system challenges. To realise improved health outcomes for Māori, the value placed on whānau and community perspectives not only needs to be acknowledged in the implementation of health interventions, health and social policies and funding arrangements, but performance measures, service design and delivery must evolve to accommodate these perspectives in practice.

## Background

Type 2 Diabetes (T2D) is a common long-term condition that significantly impacts on the health and wellbeing of New Zealanders [1, 2]. People with T2D experience increased risk of cardiovascular disease and other complications such as kidney failure, lower-limb amputations and blindness [3]. In New Zealand, it is estimated that 260,000 people have T2D, 100,000 have undiagnosed T2D, and one in every four New Zealanders is pre-diabetic, which puts them at increased risk of developing T2D and cardiovascular disease [3]. Māori (the Indigenous people of New Zealand who make up approximately 15% of the overall population), Pacific Islanders, Indo-Asians and people with a lower socioeconomic status are at an increased risk of developing pre-diabetes and T2D; similarly, there are significant inequities between Māori and non-Māori for T2D complications [2, 3].

More specifically, self-reported prevalence of T2D among Māori was about twice that of non-Māori in 2013/14 (and is most likely an underestimate of the true prevalence because some people living with T2D have not yet been diagnosed) [3]. Similarly, there are much higher inequities between Māori and non-Māori for T2D complications. For renal failure, one of the complications of T2D, rates of renal failure with concurrent T2D for Māori aged 15 and over were more than 5 times that of non-Māori at the same age group in 2012–14 [3]. While some of this difference can be attributed to the higher prevalence of T2D among Māori, the disproportionately higher rate suggests that Māori with T2D are more likely to have renal failure than non-Māori with T2D. The extent of the inequity can be estimated by dividing the relative risk of renal failure by the relative risk of prevalence, which suggests that among people with T2D, Māori are 2.8 times as likely as non-Māori to have renal failure [3]. Lower limb amputation is another complication of T2D. Similarly, rates of lower limb amputation with concurrent T2D for Māori were over 3 times that of non-Māori in 2012–14. Therefore, among people with T2D, lower limb amputations among Māori can be estimated as 1.7 times that of non-Māori [3]. With healthcare costs expected to grow, the prevention, early detection and treatment of T2D represents a serious challenge and is a priority for the New Zealand Ministry of Health.

Although modest successes have been achieved in prior T2D prevention interventions that have prioritised community engagement and cultural integration [4, 5], transformational change to the provision of disease prevention services for Māori has not yet occurred. For T2D, the lack of sustainable health change points to systemic issues that require a deeper systems action analysis of implementation pathways that engage community and culture [6, 7]. Understanding the local context for effective implementation, policy and improvement is essential. To improve Māori health outcomes, specific implementation strategies are required to ensure evidence-based interventions, while often efficacious in the research environment, also achieve the required and stated outcomes in diverse community settings within complex health systems.

The importance of stakeholder knowledge and participation in research, translation, dissemination and implementation of research findings is increasingly acknowledged [8–10]. In this way, community-based participatory research (CBPR) offers significant promise as a means to develop research that benefits the community and to achieve effective translation of research findings [11–13]. CBPR has a key focus on co-creation among community and academic partners, using culturally-centered methods, and building capacity within communities, which may be applied to improve health delivery [14–16]. Sometimes interventions that work in some communities can fail in Indigenous communities, so the He Pikinga Waiora (Enhancing Wellbeing; HPW) Implementation Framework was developed in response to common implementation challenges for interventions addressing chronic conditions [17]. The framework prioritises self-determination and consists of five elements: cultural-centeredness, systems thinking, community engagement, kaupapa Māori (an approach that is by-Māori, for-Māori and guided by Māori worldviews and principles), and integrated knowledge translation by engaging with end users. This framework is consistent with CBPR philosophy in that it emphasises a contextually based implementation of an intervention that is developed through a participatory process. However, it specifically centres Indigenous worldviews and perspectives within systems thinking which is important for sustainability and effectiveness of interventions for Indigenous communities [17–19].

Implementation challenges arise in part due to the aetiology of T2D, which comprises a complex mix of social, cultural, genetic, physiological, psychological, familial, economic, and political factors. To achieve value and high performance of the whole health system as well as its component parts, the development of an outcomes-based approach is required across connected parts of the system to guide the delivery of constantly improving health services. This is a critical issue in health systems management [20]. Currently there is a wide range of performance indicators and reporting requirements but there is an understandable tendency to measure what can easily be measured, which often concerns process and activity rather than outcome. Few indicators evaluate team work and transitions of care across sectors throughout the patient journey in a way that is consistent with integrated care or the concept of hauora. This concept is central to Māori health and wellbeing and is illustrated by the Whare Tapa Wha Māori model of wellbeing, which is applied in Kaupapa Māori and some general services and sectors. It is a holistic framework that addresses physical, mental and emotional, social and spiritual wellbeing [21]. Although a necessary part of the system, attention has often centred on performance indicators for acute hospital care rather than primary or continuing care, further limiting their clinical reach and utility [22]. Nevertheless, there is scope to widen the range and increase the utility of performance indicators, aided by the rapid expansion of information technologies in health systems [20].

Despite these opportunities and recognition of important performance indicators, there is sparse research about how to leverage these elements for addressing health inequities especially within Indigenous and Māori communities. In particular, there is limited research about how Māori (and Indigenous) health organisations view and create opportunities within the health system to address inequities. This limited research includes examination of enablers and barriers to implementing health interventions to address chronic diseases for Indigenous patients in primary care [23]. Gibson and colleagues completed a systematic review of literature in this context and found five categories of barriers and enablers: a) design attributes; b) chronic disease workforce; c) clinical care pathways; d) patient-provider partnerships; and e) access. These categories can be both enablers and barriers of implementation depending on the source (e.g., who delivers the intervention) and how they are implemented (e.g., clinical pathways). While this review did include some organisational elements in implementation, it did not explore larger systematic elements and multiple stakeholders’ perspectives, nor did it consider how to leverage these elements specifically.

To address this knowledge gap, in collaboration with a Māori health organisation responding to pre-diabetes and following the HPW Implementation Framework elements, the aim of this research was to identify strategic opportunities that may be considered and applied by the organisation, government funders, and policy makers to improve health outcomes for Māori with pre-diabetes. More specifically, the purpose of this study was to explore the questions of how the strengths of Māori heath organisations may be leveraged, and how the barriers and constraints experienced by Māori health organisations may be negotiated, for the benefit of Māori; and from a systems perspective, to identify strategic opportunities that may be considered and applied by Māori health organisations, funders and policy makers to respond more effectively to pre-diabetes and reduce health inequities between Māori and non-Māori.

## Methods

Case study methodology was utilised to explore key relationships, partnerships, contracts, funding streams, services and organisational strengths and barriers [24]. Data sources were triangulated [24] including data from semi-structured interviews and documentation to provide an understanding of the organisation. In addition, a systems map [7] was utilised as an additional approach to organise and analyse information about the complex and dynamic public health phenomenon, pre-diabetes [25].

### Document retrieval and case study context

Documentation was shared by Poutiri Charitable Trust (‘Poutiri Trust’) to provide an understanding of the organisation including a detailed history and current mission, vision and values. The primary documents that were provided by the organisation included Annual Reports and examples of contracts and key performance indicators. Further, the organisation’s web site was reviewed. An analysis of these documents provided useful background to, and an overview of, the organisation.

Poutiri Trust was established in 1997 and exists so that whānau (extended family groups), hapū (subtribes) and iwi (tribal groups) of the four waka (allied kinship groups descended from the crew of a canoe which migrated to New Zealand and occupying a set territory) – Te Arawa, Matatua, Takitimu, and Tainui – may achieve whānau ora (family health). Poutiri Trust has used Te Pae Mahutonga (a Māori health promotion framework) [26] to describe what whānau ora means to the organisation [27]; specifically: Mauriora (access to the Māori world), Waiora (environmental protection), Toiora (healthy lifestyles), Te Oranga (participation in society), Nga Manukura (leadership), and Te Manawhakahaere (autonomy).

Poutiri Trust contracts and assists to develop Māori health providers within the Bay of Plenty region of New Zealand to deliver a variety of health and wellbeing services. Poutiri Trust offers Pouwhenua clinics, working in conjunction with general practice, to provide long term condition management with the aim of reducing exacerbations and avoidable hospital admissions. As an organisation, Poutiri Trust has undergone significant change over the past two years, with changes in Board of Trustee membership at the governance level; changes in the number of staff employed directly by Poutiri Trust, the focus of key roles within the organisation; and changes in membership within the Poutiri Trust provider network. The changes occurred in response to a strategic review and external financial audits [28] and were implemented to support the sustainability of the organisation and continuous quality improvement.

### Interviews

People with a long history with the organisation and/or in key positions of leadership (governance, management and clinical) were selected for participation in conjunction with the organisation (Table 1). Key informants were interviewed until saturation was reached. Saturation occurred when the same themes were recurring, and no new insights were given by additional sources of data.

**Table 1:** Key informant demographics

Participant	Role	Ethnicity	Gender	Organisation
1	Management	Māori	Female	Poutiri Trust
2	Management	Māori	Female	Poutiri Trust
3	Clinical	Māori	Female	Poutiri Trust
4	Governance	Māori	Male	Poutiri Trust
5	Governance	Māori	Female	Poutiri Trust
6	Governance	Māori	Female	Poutiri Trust
7	Decision maker	Māori	Male	Government funding organisation
8	Decision maker	Māori	Female	Government funding organisation
9	Subcontractor	Māori	Female	Primary care provider

To field test and iteratively refine the interview questions, an experienced community researcher who is not involved in this research was interviewed, within a hypothetical context, using the research questions. Data was collected in the form of feedback and commentary about the interview schedule and minor changes were made to the interview protocol. Semi-structured format questions were used flexibly, being omitted, adapted, or elaborated according to the demands of individual context (for example, if the participant had already answered the question). Whilst trying to avoid directive or closed questions or interpretations the interviewer adopted an approach that promoted a two-way dialogue with which to explore key themes [29]. In the context of the system map, the interview explored questions about Poutiri Trust including purpose, capacity and capability, funding and partnerships, reporting performance measures, (cost of) change and organisational strategy and systems approaches (Table 2).

**Table 2:** Case study data source and analysis framework

Data source(s)	Questions	Focus of the analysis
*Documentation and interviews*: Purpose of the organisation, mission, vision, strategy	• What is the purpose of the organisation? • How does your organisational strategy help to achieve this purpose?	• Are these documents internally consistent? • Is there an alignment between these documents and the ‘current state’ of the organisation?
*Documentation and interviews:* Capability and capacity of the organisation	• What are the strengths of your organisation? • What barriers does your organisation face?	• How is the organisation structured? • What is the level of capacity and capability in the organisation to achieve its purpose?
*Documentation and interviews:* Funding and resourcing in relation to strategy: key contracts/funding	• What has been the approach to funding over time? • What has been the organisation’s response to changes in the funding streams available over time?	• Develop a map of current contracts and funding streams. • What are the current strategic relationships (with providers, funders, other partners) at an organisational level.
*Documentation and interviews:* How could the organisation operate more effectively in the system? Change and cost of change	• What sort of shifts are difficult for the organisation to cope with? • Have shifts in external funding streams been linked to performance? • What has been the impact of these changes in relation to equity?	• Key relationships and contracts for the organisation. • When organisations are forced to be opportunistic in relation to funding opportunities this can result in a significant ‘cost of change’. Have any changes in funding/direction been ‘evidence-based’? • How could the system support organisations to be more effective?
*Documentation and interviews:* Measurement, reporting, performance and role of organisation	• How does the organisation know if it is achieving its purpose? • Are the metrics specified by funders or the organisation effective measures of performance?	• Are organisations ‘policy takers’ or do they influence the system? • Are there opportunities for organisations to better utilise data to more effectively influence the system and/or guide funding decisions?
*Documentation and interviews:* Organisational strategy	• Does the organisation focus specifically on pre-diabetes, or is effort in this area included as part of a broader focus on long term conditions, determinants of health, or another planned strategy?	• Is there evidence of an emergent, opportunistic approach or a planned strategic approach to service provision? • Are the benefits of a longer-term strategy and the ability to align resources (including relationships) evident, including systems approaches?

**Fig. 1:**
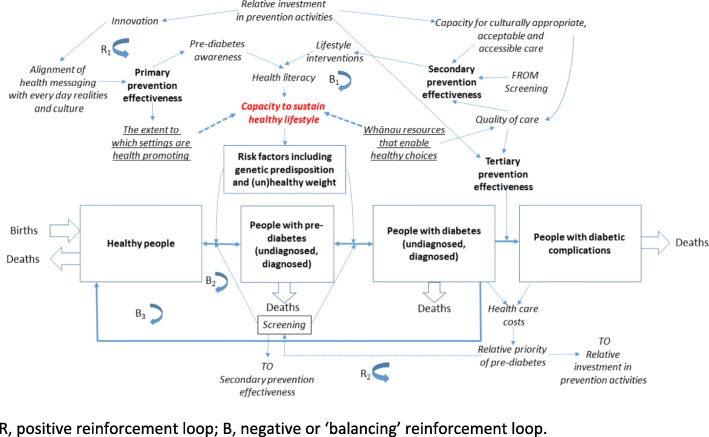
Systems map [7]

Systems thinking (especially system dynamics) has been applied to various public health issues including diabetes, childhood obesity, asthma, tobacco control, cardiovascular disease and family violence prevention. This is a relatively novel but useful approach within public health. A systems map [7], was utilised in this case study (Fig. 1) as an additional approach to consider strategic opportunities available to Poutiri Trust.

Prior to completing the interview, all participants were invited to read an information sheet about the study, to clarify any concerns or questions, and to sign a consent form before being interviewed and recorded for between 45 and 90 min. Ethical approval for the study was provided by the review board of the Waikato Management School. The interviews were completed at a place of safety for both participant and interviewers and usually included an office.

The interviews and documents retrieval was completed by a member (AB) of the larger research team who was not directly working with the community organisation. This particular case study was conducted prior to the initiation of a co-designed health intervention to address pre-diabetes and related conditions. The lead researcher thus has a role as non-participant researcher as did two other co-authors (MH & JF). The other co-authors (JT, CM, JO) were members of the team directly constructing the health intervention. This approach provided a balance of “objective” and “subjective” perspectives in the data analysis, while mitigating bias during the data collection process.

### Data analysis

A guided thematic analysis [29] was conducted across the data set, giving full and equal attention to each data item, to identify and analyse repeated patterns of meaning (themes) within the data generated from interviews and documentation across six domains – purpose, capacity and capability, funding and partnerships, reporting performance measures, (cost of) change and organisational strategy. Transcripts of interviews and documents (Phase 1: Data familiarisation) were read in their entirety and in an active way to identify repetition, recurrence and forcefulness or words, phrases, or themes (termed ‘concepts’) that responded to the key research aims/questions:

- What are the organisational strengths and resources that may be applied to respond to pre-diabetes?
- What are the organisational barriers and constraints that need to be addressed to respond to pre-diabetes?

A coding scheme was developed containing concepts (and subsidiary concepts), and their definitions (Phase 2: Generating initial codes). As the analysis progressed, some concepts were modified to ensure they conveyed the meaning the participants had expressed in the interviews, and supporting direct quotes were identified in the data. The process of coding was utilised to organise the data into meaningful groups, which were organised under broader themes (Phase 3: Searching for themes). The relationship between codes, between themes, and between different levels of themes was also considered as the themes were reviewed (Phase 4: Reviewing themes) and refined (Phase 5: Defining and naming themes). Findings were checked with case study participants to enhance validity [30].

## Results

Several themes and opportunities were identified, which may be addressed to create a system that better supports Māori organisations to realise health gains for Māori [31]. Specifically, the need to: a) recognise and leverage the strengths of Māori health organisations, as conduits for the community voice and influential partners in the community to effect change; b) strengthen partnerships for community benefit to support quality service delivery and measurable, evidence-based change that matters to communities; and c) recognise the importance of hauora and support intersectoral integration of health and social services to enhance whānau-centred care and more equitable service delivery.

### Māori health organisations are conduits for the community voice

Māori health organisations are integral to communities, which makes them ideal conduits for the community voice and influential leaders to effect change, to promote community engagement and to ensure consideration of the local context. There is increasing pressure on all organisations to leverage organisational data for reporting purposes; to demonstrate quality service provision and improved health outcomes. As funder expectations increase in this regard, for many organisations this represents a significant capability and capacity challenge. There is some risk that existing health inequities, including those relating to diabetes, may be exacerbated if non-governmental Māori organisations do not successfully negotiate these challenges.

In a health system that requires improved capacity for culturally appropriate, acceptable and accessible care, it will be important to support capability and capacity building for Māori organisations to leverage data to shift organisation-level performance, report on continuous quality improvements and exert influence within the health system to deliver change that matters to communities.

With today’s technological advancements there is a lot that clinicians can tell about a patient without even talking to them (for example, their physiological parameters and recent admission history). However, unless patients and their whānau are asked what is important to them and how they rate their quality of life and experiences of the health care and services provided, clinicians do not actually know the whole picture [32, 33]. Staff member from a government funding agency confirmed this was the prevailing approach when setting contract key performance indicators: “*We’ve got clinicians who advise us on what measures should be included in contracts. And I think we tend to rely heavily on them when it comes to things like these [long term] conditions.”* (Staff member 2, Government funding agency). The need to adopt a different approach that expands beyond (largely) clinical indicators, to include quality of life and experiences of care was viewed as important by those interviewed, who felt this would ensure that measures of success are more inclusive and consistent with Te Ao Māori, a Māori worldview. A board member reiterated this point: “*Because health and wellbeing to Māori is not just what health is to the health system. [Contracts] need to reflect that, because my understanding is historically contracts have been health focused but not Māori health focused.”* (Board member 2, Poutiri Trust).

Furthermore, this would support Poutiri Trust to demonstrate a wider range of positive outcomes back into the system in a way that is more consistent with the concept of hauora. Therefore, building capability to determine more effective outcome measures will be important for all stakeholders. As an integral part of this, there is scope for Māori organisations to utilise and further develop sets of outcome measures that demonstrate a wider range of positive outcomes that matter to Māori communities, which may be utilised in a range of ways, including to evidence how Māori organisations have effectively responded to community need, and to support contract procurement.

### Partnerships for community benefit

While gaps between evidence and decision-making exist in all areas of the health system [34], for Māori communities, inequitable access to the best available evidence and care is exacerbated by resource constraints [35]. Strategic partnerships may be a way to overcome this challenge and offer the opportunity to address key capability gaps by partnering with organisations who have complementary core skills. Two key aspects to a partnership approach emerged from the data, including the possibility of working with iwi (tribes) within the region, and alliancing with other Māori and non-Māori providers to secure larger, more strategic contracts. A strategic approach to collaboration and partnerships was evident, which is important to close the gap between available evidence and decision making, and to exert influence within the system. Two of the board members offered quotes that support this perspective about strategic partnerships:

*I think partnerships are essential. Relationships are essential… it’s the whole whānau collective thing. It all makes sense, collaboration makes sense. And I don’t think it can be done without that.* - Board member 2, Poutiri Trust

*It’s… about creating this network of highly motivated people, passionate people, that can walk in both worlds – that can walk in the academic side and on the ground in the community and understand people’s side of things as well and can help us develop what these programs will look like.* - Board member 1, Poutiri Trust

Furthermore, as the political landscape within New Zealand changes with more iwi settlements occurring over the coming months and years (government settlements with tribes based on the founding treaty of New Zealand), there may be opportunities for pan tribal organisations to work in partnership to advance more explicitly the specific vision and health goals of individual iwi. One of the Poutiri staff members noted this possibility:

*But Iwi settlements, where Iwi are becoming more established in their own rights and so less likely to collaborate anymore to come together as a single voice... Some of them want to get into the health space… We all have links with different Iwi but we don’t have a process to engage with Iwi to have those discussions.* - Staff member 3, Poutiri Trust

The potential for larger scale ‘alliancing’ was also discussed by funding agency staff, as a mechanism to build local capacity and to secure larger service contracts within the region, which is a strategic approach that may be considered by Māori organisations. This approach allows for Māori health providers to work with larger national providers, and non-Māori providers in the region.

*… the bigger providers don’t necessarily have the reach into these communities. … and they don’t know the stories that happen, so they can learn from our providers but then some of our little providers or some of our providers can actually learn from the systems that national companies have.* - Staff member 1, Government funding agency

In sum, partnerships and alliances have the potential to offer benefits for all organisations involved and the communities they serve. For example, at the organisation level, it may be possible to make joint bids for larger contracts by strategically selecting who to work with on the basis of core organisational skills, strengths and values. Collaborative working relationships between a wider range of health professionals and health and social services, including non-Māori providers, may lead to more culturally acceptable, accessible, integrated care in the region.

### Integrating health and social services for whānau-centred care

Contracting is a mechanism to clarify roles and create accountability as well as align goals between government agencies and providers through appropriate incentives. It has been used as an attempt to meet a variety of aims, including to improve outcomes, lower costs, increase coverage of and access to services, improve service quality, and improve efficiency of resource utilisation [36]. Unfortunately, contracts may also have a short-term focus, lead to perverse incentives, may stifle innovative providers, lead to duplication across funding agencies, and result in high reporting loads and compliance costs for providers [36]. Indeed, the scope and structure of current primary care contracts and the way performance is currently measured makes it difficult at times to provide fully funded integrated care that is consistent with the concepts of hauora and client-centred care, which is central to the mission and vision of many Māori organisations. One staff member mentioned the ideal approach when referencing contracts: “*We need to work around the client, not around [our] contracts.”* (Staff member 3, Māori organisation).

Nevertheless, participants reported that New Zealand government agencies are increasingly taking an inter-agency approach to health and wellbeing, which theoretically provides a mechanism for organisations to provide fully funded, more holistic care in a way that acknowledges the link between health outcomes and the social determinants of health. For example, a government funding agency staff member noted support for this approach: “*[It] is an approach that DHBs (District Health Boards) are really looking into – how can we work more collectively as multi-agencies? … that’s exciting for that integrated care and whole person kind of approach.”* (Staff member 1, Government funding agency). The second funding agency staff member built on this perspective by providing a concrete example of integrated care:

*… we understand that there are a lot of players in health that contribute to the wellbeing of the community …we’ll get alliances through other different inter-agency groups; seeing how we can leverage their resources to help support, say for example, respiratory conditions… we obviously need to partner with housing.* - Staff member 2, Government funding agency

Pre-diabetes is currently being addressed by many Māori organisations as part of a broader focus on long-term conditions and a more holistic approach to health and wellbeing; although, the need for a clinical response to pre-diabetes was also seen as important because lifestyle interventions (including diet and exercise) were not always observed to be effective without the appropriate support for individuals and whānau (extended family or community of individuals). Both board and staff members of Poutiri Trust offered views aligning with this perspective. For example, a board member stated,

*It would be my hope that it was more around chronic disease prevention and management, not just one condition. It doesn’t make sense in a Māori world to think about “a condition” and “b condition”. The one thing I do like about [the focus on] prediabetes is there is a prevention focus.* - Board member 2, Poutiri Trust

In sum, an effective response to T2D will require of the ‘big picture’ promotes effective interactions across sector and organisation boundaries. This requires recognition of multiple perspectives and world views, for which Māori organisations are conduits. For most non-governmental organisations, funding sources are increasingly insufficient to meet growing health and social needs and rising costs. This makes it very difficult for these organisations to undertake long-term planning, improve their services and reach their full potential. This deserves the attention of policy makers and funders, who also have a vested interest in seeing Māori organisations continue to grow and prosper.

## Discussion

Colonial processes have undermined Māori social, economic and political structures over time, resulting in redistribution of power and resources in favour of non-Maori, which is reflected in health inequities [37]. There is a risk that inequities will be perpetuated as the health system scrambles to reduce the prevalence of, and complications associated with, T2D. As part of the solution, there is a need for Māori organisations to leverage their community connectedness and other organisational strengths, as a key mechanism for enabling self-determination and innovation [38]. This section discusses the implications of the study findings in the context of the extant literature also noting limitations and conclusions.

### Māori health organisations are central to implementation success

Strengthened partnerships with Māori health organisations will support measurable, evidence-based change and service delivery, including in relation to pre-diabetes and underlying social determinants of health, particularly when Māori knowledge systems are viewed alongside a Western scientific approach, which from a systems perspective requires alignment of health messaging with every day realities and culture.

Systems thinking facilitates new strategies and actions by considering multiple viewpoints within a ‘system’, and the interactions within and across organisational boundaries required to produce better outcomes [39]. In this way, greater recognition of the strengths of Māori organisations – as partners to effect change, promote community engagement and ensure consideration of the local context – is an important factor for the successful implementation of pre-diabetes interventions and services, and to ensure that the implementation of innovations do not unwittingly increase inequities [31]. Centring Māori perspectives and valuing community voice represents a promising approach to achieving improved health equity for pre-diabetes and diabetes, and Māori organisations are well positioned to work with other Māori (and non-Māori providers) to ensure this occurs. Recent research suggests that integrating systems thinking with Indigenous perspectives holds promise for health interventions addressing obesity for Māori [20].

Indeed, drawing on work to expand indicators beyond the (largely) clinical domain may assist with this goal. Evidence demonstrates that clinical indicators (like biomarkers) often fail to correspond with how a patient is actually feeling, further demonstrating the importance of routine and timely collection of patient’s perceptions of their health and wellbeing [32]. This can be achieved through the systematic collection and use of Patient Reported Measures (PRMs) (Patient Reported Outcome Measures, PROMs; and Patient Reported Experience Measures, PREMs) as an integral part of an overall reporting framework. There is good evidence to demonstrate that patients who are more engaged in their healthcare tend to choose less costly interventions (e.g. presenting to a physiotherapist for lower back pain instead of hospital emergency) [33]. PRMs have also been well documented to support clinician decision making, shared care planning and are a good indicator for overall patient outcomes; especially in those conditions marked by morbidity rather than mortality [32, 33].

While performance measures commonly prioritise a clinical perspective over the patient- and whānau-perspective, there is potential for Māori organisations to work with government funding agencies to co-design for existing and new contracts measures that are more meaningful for patients and whānau. Indeed, any attempt to measure value in health care must incorporate patient perspectives [33]. This is one example of how Māori organisations may exert more influence within the health system. More generally, it was the perception of those interviewed that Māori organisations have limited opportunity to feedback into the health system although, a clear aspiration to do so was expressed. Such an approach is consistent with the extant literature on CBPR for Indigenous and other communities to facilitate self-determination, ownership and sustainability of health interventions [14–17].

To highlight the role of Māori health organisations as critical actors within the health system, the key findings and strategic opportunities identified are summarised in Table 3 (column 1) and are aligned to key health system challenges (column 2); how funders and policy makers might act to better position Māori organisations to overcome these challenges (column 3); and how key elements of the HPW implementation framework provide a possible mechanism to strengthen leadership by Māori health organisations and thereby reduce health inequities (column 4). Continued and coordinated effort by all actors is needed to improve and protect the health of populations, with a focus on reducing inequities.

**Table 3:** Summary of findings and recommendations of relevance to Māori organisations, and funders and policy makers

Opportunities for Māori organisations to reduce health inequities	Current implementation challenges within the wider health system, which contribute to health inequities	Recommendations for consideration by funders and policy makers to support the work of Māori organisations	Benefits of addressing implementation challenges with the aim of reducing inequities
Māori health organisations are conduits for the community voice and are influential partners in the community to effect change	Lack of Cultural Centredness and Kaupapa Māori (KM) approach – communities have, to date, had limited involvement in defining the ‘problem’ or developing the ‘solution’	Leverage connections with communities by demonstrating greater recognition of the strengths of Māori organisations as partners needed to effect change, promote community engagement and ensure consideration of the local context	The community voice is heard to ensure the local context is understood and to promote greater community engagement, participation, and control; and better ‘alignment of health messaging with everyday realities and culture’ (Fig. 1).
A strategic approach to partnerships for community benefit will support Māori health organisations to deliver high quality services and evidence-based change that matters to communities	Poor integrated knowledge transfer - Health services are placed in the community with no consultation, and the community does not trust or have the required level of comfort with the service	Demonstrate strengthened partnerships between government agencies and Māori organisations to support measurable, evidence-based change and service delivery that matters to communities	Improved integrated knowledge transfer, facilitated by Māori organisations and their partners. Knowledge users realise the benefits of evidence-based change, which aligns with a KM approach and Indigenous self-determination; and improved ‘capacity for culturally appropriate, accessible and acceptable care’ (Fig. 1).
Recognise the importance of hauora and support an inter-sectoral approach for health and social service integration that supports Māori health organisations (and others) to deliver whānau-centred care	Limited importance has been placed on Systems Thinking – despite the importance of hauora and the delivery of whānau-centred care, it is challenging for providers to deliver integrated health and social services	Provide more seamless, integrated planning and funding across government agencies to support integration of health services with services that, at least in part, influence the social determinants of health	An appreciation of the ‘big picture’ promotes effective interactions across sector and organisation boundaries. This requires recognition of multiple perspectives and world views, for which Māori organisations are conduits; and the ‘extent to which settings are health promoting’ (Fig. 1).

### Implementation to reduce health inequities for indigenous communities

The HPW Implementation Framework was developed to address common health service and intervention implementation challenges for Indigenous communities [17]. The framework is intended to be used as a planning tool for funders and policy makers to guide effective implementation of services and innovative interventions. Funders can use the framework to assess the likely effectiveness of proposed services, interventions, and research. This underscores the importance of Māori health organisations, who are well placed to develop and implement effective services and interventions targeting the prevention and management of long-term conditions such as diabetes. This approach along with the findings of the current study helps to extend the research on implementation enablers and barriers for primary care interventions for Indigenous patients [23]. The current research illustrates the complexity of systems and funding structures in addition to workforce and cultural elements associated with implementation of interventions.

Currently, the level of funding and nature of the contracts held by Māori health organisations raises questions about the relative value placed on preventing pre-diabetes, particularly given the importance of ensuring primary care provision is culturally appropriate, acceptable, and accessible [7]. Nevertheless, taking a kaupapa Māori approach was considered by funders in the current study to be important to achieve improved health outcomes for Māori, at least in principle. With the support of funders, Māori organisations are well positioned to provide kaupapa Māori services and initiatives that promote greater community engagement, participation, and control for implementation that results in improved health outcomes for Māori consistent with the HPW framework [34, 40, 41]. This would be enhanced by supporting capacity and capability building to occur in Māori organisations and to make organisation-level and system-level improvements. If not, there is a risk that inequities will be further exacerbated.

To take an approach that is consistent with the concept of hauora and to deliver whānau-centred care that particularly addresses chronic and related conditions, inter-sectoral integration of health and social services is required [42, 43]. Scholars argue that integrated care is a key method for addressing health inequities [44]. Participants suggested that this must be supported by more seamless funding across government agencies, an appreciation of the ‘big picture’ facilitated by systems thinking, and support for organisations who interact across sector and organisation boundaries to produce better outcomes. This can only be achieved when value is placed on recognising multiple perspectives and worldviews within the system [19] and more explicit links are made between funding streams for health and other services or initiatives that influence the social determinants of health and the local environment.

### Limitations

This case study is intended to capture the complexity of responding to pre-diabetes and the associated health inequities within the health system, which can be difficult to represent simply and is therefore a limitation of this approach. Similarly, although this case study cannot be representative, it can inform situations and approaches beyond the actual case that was studied. Therefore, this research was designed to provide detailed insight and in-depth data into the role of Māori health and/or Indigenous health organisations, who commonly face many similar challenges in their work to reduce health inequities, and is useful to understand complex inter-relationships between the qualitative data and to inform policy. Nonetheless, the focus on a single case organisation does have limits in understanding the larger healthcare system in the context of health inequities for Māori. Future research can consider the interplay of multiple organisations in the context of constructing integrated care models and implementing interventions for pre-diabetes and related conditions.

## Conclusion

Māori health organisations are important actors in the prevention of diabetes as they have influence within their communities with potential to link with marae (traditional meeting places), kura (schools), sports clubs, regional councils, urban planning functions and workplaces and other stakeholders effectively working across sectors to make ‘environmental’ changes that reduce the risk factors for several long-term conditions. The concept of hauora is of relevance when considering the overall approach to pre-diabetes and diabetes and what constitutes value in relation to the provision of health services. A primarily clinical approach to pre-diabetes can downplay the significance of social, cultural, economic, and political factors, especially because (un)healthy weight is a risk factor that is shared with diabetes and various other chronic conditions. For Māori organisations, this aligns with the approach to pre-diabetes and long-term conditions to date, which is consistent with the concept of hauora. Consideration by policy makers of how this approach may be supported by, and reflected in, funding streams warrants further attention.
